# Key messages: Complicated cataract surgery

**Published:** 2019-02-10

## Initial consultation

**Figure:**
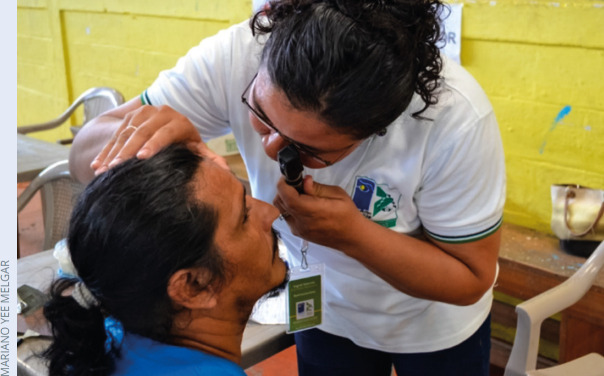

- Identify patients in whom cataract surgery will be complicated or difficult; this avoids unwelcome surprises in the operating theatre
- Manage patients' expectations. Explain what to expect after surgery (in terms of the visual outcome and the potential need for further treatment) and obtain informed consent

## Be prepared

**Figure:**
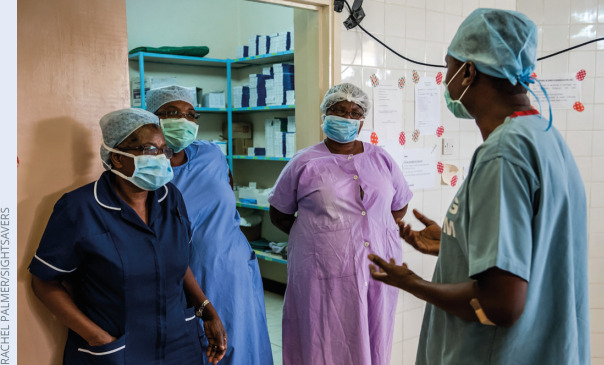

- Anticipate potential difficulties
- Check that all equipment, instruments and consumables are sterilised and available
- Brief the surgical team thoroughly

## After surgery

**Figure:**
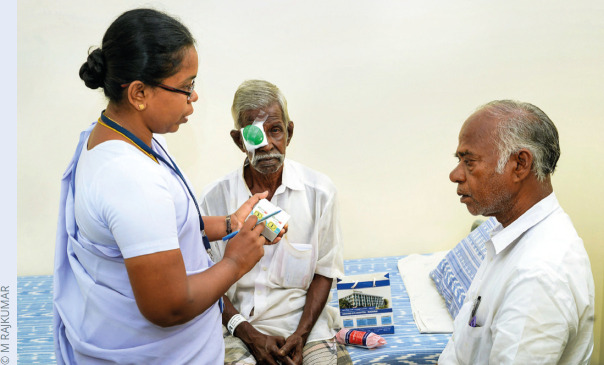


The success or failure of the operation may depend on good postoperative care.

- Ensure that inflammation is under control
- Explain to patients how important it is that they take their postoperative medication and come back for follow-up visits

